# Unlocking Mangiferin: A Therapeutic Candidate Revolutionizing Liver Disease Therapy

**DOI:** 10.3390/nu17213401

**Published:** 2025-10-29

**Authors:** Jihang Xie, Sijing Su, Jianfa Wu, Xing Yang, Qian Zhang, Xiaojiang Shen, Linlin Zhao, Ting Wang, Nana Feng, Jinsong Su, Yi Zhang

**Affiliations:** 1 State Key Laboratory of Southwestern Chinese Medicine Resources, College of Pharmacy, Chengdu University of Traditional Chinese Medicine, Chengdu 611137, China; xiejihang11@stu.cdutcm.edu.cn (J.X.); wujianfa0626@163.com (J.W.); zhangqian7412@stu.cdutcm.edu.cn (Q.Z.); fengnana@stu.cdutcm.edu.cn (N.F.); 2 School of Ethmic Medicine, Chengdu University of Taditional Chinese Medicine, Chengdu 611137, China; 18288627707@163.com (S.S.); yangxing19970914@163.com (X.Y.); zhaolinlin0214@stu.cdutcm.edu.cn (L.Z.); wangting19981213@163.com (T.W.); 3 Fujian Key Laboratory of Polymer Materials, Fujian Provincial Key Laboratory of Advanced Materials Oriented Chemical Engineering, College of Chemistry and Materials Science, Fujian Normal University, Fuzhou 350007, China; sxj991118@163.com; 4 Innovation Institute of Chinese Medicine and Pharmacy, Chengdu University of Traditional Chinese Medicine, Chengdu 611137, China

**Keywords:** mangiferin, therapeutic candidate, hepatoprotection, oxidative stress, biosynthesis and chemical synthesis, bioavailability

## Abstract

Mangiferin (MF), a natural component widely found in fruits, vegetables, and herbs, has garnered increasing attention for its potent antioxidative properties and therapeutic potential. This bioactive xanthone compound plays a crucial role in mitigating oxidative stress, which is a key factor in the pathogenesis of various diseases, including liver diseases. As a powerful natural antioxidant, MF exhibits a wide range of hepatoprotective effects, making it a promising candidate for liver disease therapy. In this review, we systematically examine the source and chemical properties, synthetic pathways, pharmacokinetic characteristics, and bioavailability enhancement strategies of MF. Furthermore, we explore its mechanisms of action in treating liver diseases, with a focus on its antioxidative properties and their role in modulating liver disease progression. Given the growing burden of liver disease and the limitations of current therapies, this review aims to promote the clinical application of MF as a therapeutic candidate, paving the way for innovative therapeutic strategies for liver diseases.

## 1. Introduction

Mangiferin (MF), a bioactive xanthone compound widely found in a variety of fruits, vegetables, and herbs, has attracted growing interest due to its remarkable antioxidative effects and therapeutic versatility. The core pharmacological feature of MF lies in its potent broad-spectrum antioxidant activity. By precisely regulating tissue-specific antioxidant signaling pathways, it provides comprehensive protection against damage to the kidneys, cardiovascular system, nervous system, skeletal system, reproductive metabolic system, and diabetes-related organs. For the kidneys, it offers antioxidant protection against diabetic nephropathy, renal ischemia–reperfusion injury, and hypertensive nephropathy [1,2,3]. In the cardiovascular system, it counteracts myocardial apoptosis [4]. In the nervous system, it ameliorates depression, neuronal injury, and Parkinson’s-related damage [5,6,7]. It also protects bones, alleviates oxidative damage in polycystic ovary syndrome [8,9], and supports protection of diabetes-related organs [10]. This multidimensional mechanism has been thoroughly validated. This compound plays a critical role in counteracting oxidative stress, which is a key contributor to the onset and progression of numerous diseases, including liver disorders. The unique structure of MF, characterized by its glucosyl-modified xanthone skeleton (C2-β-D-glucoside), provides the molecular basis for its ability to modulate multiple cellular pathways, offering a multi-target approach to therapeutic intervention. Recent studies have demonstrated that MF can alleviate lipotoxicity, restore redox balance, inhibit pro-fibrotic signaling, and block tumor cell cycles, positioning it as a promising pleiotropic hepatoprotective agent in the context of liver disease [11,12,13,14].

Liver disease, one of the most pressing global health concerns, continues to rise, with viral hepatitis, alcoholic liver disease (ALD), and metabolic-associated fatty liver disease (MAFLD) leading to millions of deaths annually. Despite significant advances in the understanding of liver pathology, current treatments remain limited, with antiviral drugs, immunomodulatory therapies, and the development of drugs for metabolic liver diseases still facing challenges. ALD, for example, lacks approved therapies, and although Resmetirom will soon be approved for MAFLD, the overall number of newly approved liver disease drugs remains low across the US, China, Japan, and the EU. The urgent need for multi-target, low-toxicity therapies is critical [15].

In this context, the antioxidant and hepatoprotective properties of MF offer a promising avenue for innovative treatments targeting the oxidative mechanisms underlying liver disease pathology. Its ability to target multiple pathways involved in liver injury, including oxidative stress, inflammation, and fibrosis, positions it as a leading candidate for further clinical exploration. However, its clinical application has been limited by challenges typical of natural products, such as low oral bioavailability and poor metabolic stability. To overcome these obstacles, ongoing research is focused on enhancing its pharmacokinetic profile through structural modifications, novel drug delivery systems, and biotransformation strategies [16,17,18,19].

This review aims to synthesise recent research advances concerning the chemical properties, biological activity, pharmacokinetics, and structural modifications of MF, with particular emphasis on its translational significance as a nutritional supplement in the treatment of liver disease. By examining its molecular mechanisms and potential applications in liver disease, this paper provides a theoretical foundation for the development of precision therapies based on natural products, with a particular focus on the antioxidant properties of MF and their role in liver disease intervention.

## 2. Method

We conducted literature retrieval of MF using electronic databases, including PubMed, CNKI, Web of Science, SpecialSciDBS, Elsevier, and academic papers, to conduct a comprehensive analysis and summary. We used mangiferin, pharmacological activity, hepatoprotection, oxidative stress, bioavailability, antioxidant, biosynthesis and chemical synthesis as keywords to review the information about source, phytochemical properties, synthesis, pharmacokinetics, pharmacology analysis and bioavailability studies of MF.

## 3. Source and Phytochemical Properties of MF

**Figure 1:**
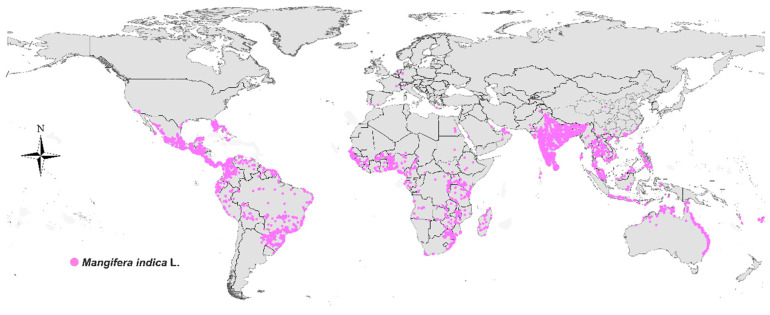
The global distribution of mangoes.

**Figure 2:**
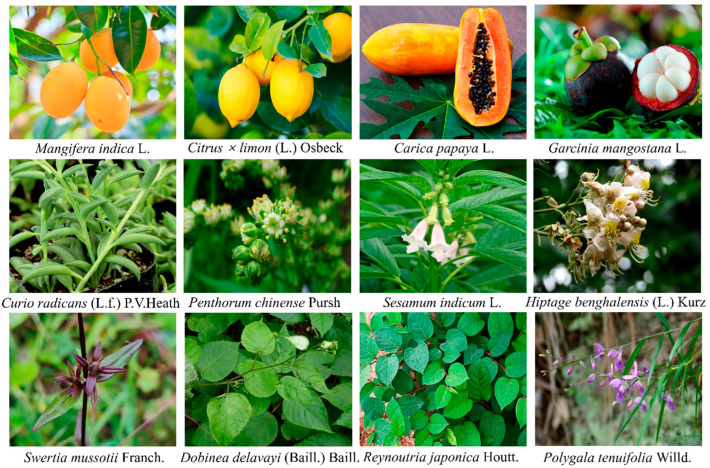
Some species containing mangiferin. Species images are reproduced from the POWO (Plants of the World Online) website (https://powo.science.kew.org/) with permission from [Royal Botanic Gardens, Kew], copyright [2025] and reproduced from the GBIF (Global Biodiversity Information Facility) website (https://powo.science.kew.org; https://www.gbif.org/) with permission from [www.GBIF.org], copyright [2025].

MF is a natural polyphenolic xanthone with a remarkably broad botanical distribution. It has been identified in over 180 species spanning more than 51 plant families, encompassing ferns, monocotyledons, and dicotyledons. The compound is not uniformly distributed across the plant kingdom but is particularly concentrated in several key families. Among these, the *Gentianaceae*, *Iridaceae*, and *Anacardiaceae* families are recognized as major sources. Notably, within the *Gentianaceae* family, certain species such as *Gentianella caucasica* have been reported to contain MF levels as high as 2.34% in their dried aerial parts. The mango tree (*Mangifera indica* L.), a member of the *Anacardiaceae* family, is another rich reservoir, with the highest concentrations typically found in the bark, leaves, and fruit peel (Figure 1). Furthermore, MF is a principal bioactive component in the rhizome of *Anemarrhena asphodeloides*, a plant widely used in traditional medicine. It is important to note that the MF content in any given plant is highly variable and influenced by factors such as the species, genetic cultivar, specific plant organ, and the methods of extraction and analysis. This diverse and widespread occurrence in the plant kingdom underscores its significance as a readily accessible phytochemical for pharmacological research and development [20,21,22,23] (Table 1 and Figure 2).

MF, a natural polyphenolic compound with the molecular formula C_19_H_18_O_11_, is a xanthone derivative [24,25,26]. Its core structure contains multiple reactive hydroxyl groups (notably at the C-2 and C-4 positions) and a glucose moiety linked via a *β*-glycosidic bond at the C-7 position. This compound appears as a yellow-to-light-brown crystalline powder, slightly soluble in water but readily soluble in polar solvents such as methanol and ethanol, with a melting point of 271~273 °C [27,28,29,30,31]. Its chemical stability is highly pH-dependent: under acidic conditions, the glycosidic bond undergoes hydrolysis to release the aglycone norathyriol [32,33,34]. Additionally, it is sensitive to light and heat, requiring storage in dark, low-temperature conditions to minimize oxidative degradation [35,36,37,38]. MF can be identified through colorimetric reactions and spectroscopic analysis (characteristic UV absorption peaks at 258 nm and 316 nm) [39,40,41]. Its polyhydroxy structure confers strong antioxidant activity, enabling free radical scavenging and metal ion chelation. However, while the glucose moiety enhances water solubility, it also limits bioavailability [23,42,43,44,45].

**Table 1:** Sources of MF [23,41].

No.	Scientific Name	Common Name	Source Parts in Plants
1	*Mangifera indica*	Mango	Fruits, leaves, bark
2	*Mangifera persiciformis*	Peach-shaped Mango	Fruits, leaves, bark
3	*Mangifera sylvatica*	Forest Mango	Fruits, leaves, bark
4	*Hiptage benghalensis*	Hiptage	Leaves; Flowers
5	*Mammea americana*	Mammee Apple	Branch; Leaves
6	*Carica papaya*	Papaya	Leaves
7	*Citrus limon*	Lemon	Fruit peel
8	*Terminalia chebula*	Chebulae Fructus	Fruit pulp
9	*Cydonia oblonga*	quince	Fruits; Seeds
10	*Prunus amygdalus*	European plum	Fruits
11	*Garcinia mangostana*	Mangosteen	Fruit peel
12	*Coffea arabica*	Arabian coffee	Leaves
13	*Phaleria capitata*	-	Seeds
14	*Phaleria macrocarpa*	-	Seeds
15	*Aspalathus linearis*	Rooibos	Leaves
16	*Gypsophila pacifica*	-	Roots; Leaves
17	*Curio radicans*	-	Roots; Stem
18	*Penthorum chinense*	all-grass of Chinese Penthorum	Leaves; Fruits
19	*Swertia macrosperma*	-	Whole herb
20	*Swertia mussotii*	Herba Swertiae Mussotii	Whole herb
21	*Swertia punicea*	-	Whole herb
22	*Swertia kingii*	-	Whole herb
23	*Swertia franchetiana*	-	Whole herb
24	*Gentianella turkestanorum*	-	Leaves
25	*Gentiana algida*	Alpine Gentian	Leaves; Flowers; Whole herb
26	*Gentiana rhodantha*	-	Leaves; Flowers; Whole herb
27	*Hedysarum alpinum*	-	Rhizome; Leaves
28	*Hedysarum flavescens*	-	Rhizome; Leaves
29	*Astragalus membranaceus*	Astragali Radix	Rhizome; Leaves
30	*Anemarrhena asphodeloides*	Anemarrhenae Rhizoma	Rhizome
31	*Aquilaria sinensis*	Chinese Eaglewood	Resin-containing heartwood
32	*Aquilaria crassna*	Eaglewood	Resin-containing heartwood
33	*Sesamum indicum*	sesame	Seeds
34	*Dobinea delavayi*	-	Rhizome
35	*Reynoutria japonica*	Giant Knotweed Rhizome	Rhizome; Leaves
36	*Polygala tenuifolia*	Thinleaf Milkwort Root-bark	Roots; Stem; Leaves

## 4. Extraction and Separation Methods of MF

The extraction and separation methods for mangiferin have developed into a relatively mature process system. Common extraction strategies include solvent extraction (such as ethanol-water systems) combined with physical assistance technologies like ultrasonication and microwaving to enhance efficiency. For raw materials with high gum content, such as mango leaves, pretreatment steps like ester extraction for impurity removal are often employed. During the separation and purification stage, macroporous resin chromatography serves as an effective method for obtaining high-purity products, often supplemented by crystallization techniques (such as recrystallization exploiting solubility differences in hot and cold methanol) for final refinement. The integrated application of these methods enables the efficient extraction of high-purity mangiferin from diverse plant sources including mango leaves and mango peel [38,39,45].

## 5. Synthesis of MF

### 5.1. Biosynthesis

**Figure 3:**
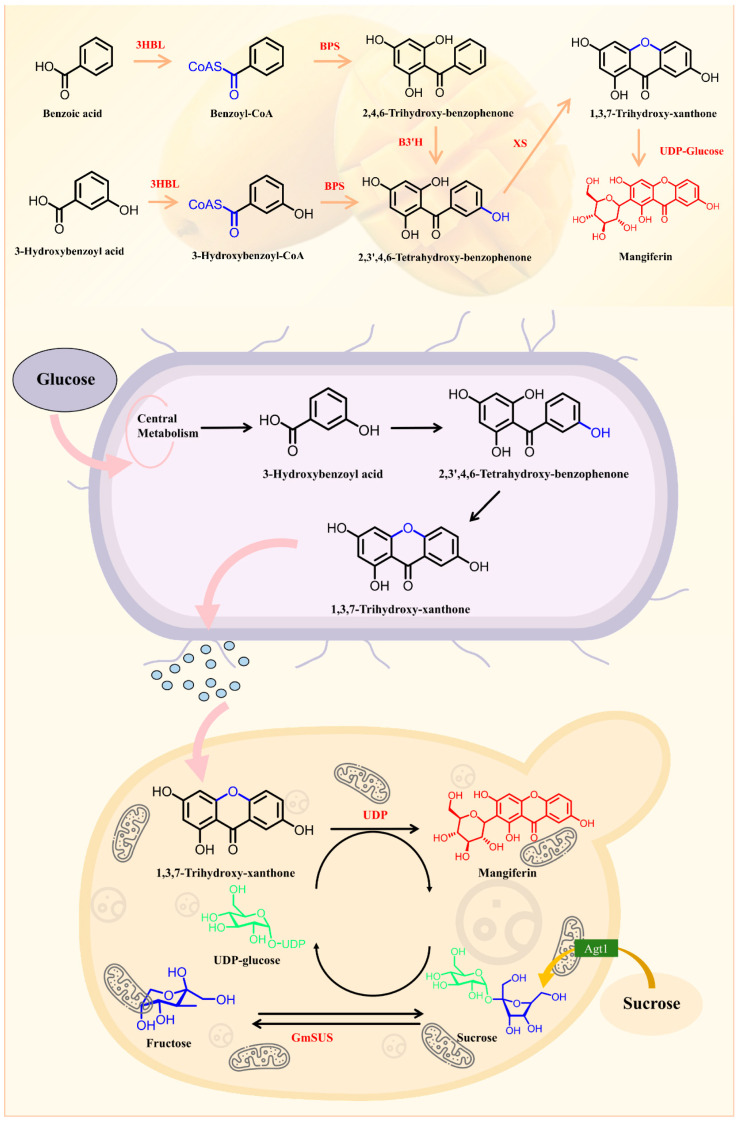
Mangiferin biosynthesis and heterologous synthesis hypothetical pathway.

MF, a bioactive xanthonoid with antioxidant and anti-inflammatory properties, is biosynthesized through the integration of phenylpropanoid and polyketide pathways. Starting from phenylalanine, phenylalanine ammonia-lyase generates p-coumaric acid, which is converted to benzoyl-CoA. A type III polyketide synthase then extends the polyketide chain using malonyl-CoA, followed by cyclization and oxidation via cytochrome P450 enzymes to form the xanthone core. Subsequent hydroxylation and methylation modify the skeleton, while a UDP-glycosyltransferase attaches glucose to the C-2 hydroxyl group to complete glycosylation. The synthesis is regulated by developmental stages, environmental stresses, and jasmonic acid signaling. Heterologous expression of key enzymes enables metabolic engineering for production, though challenges remain in pathway elucidation, enzyme activity optimization, and intermediate toxicity. Deciphering these mechanisms holds promise for natural drug development and synthetic biology applications [44,46] (Figure 3).

### 5.2. Chemosynthesis

#### 5.2.1. Total Synthesis of MF

##### Synthesis of the Xanthone Moiety

**Figure 4:**
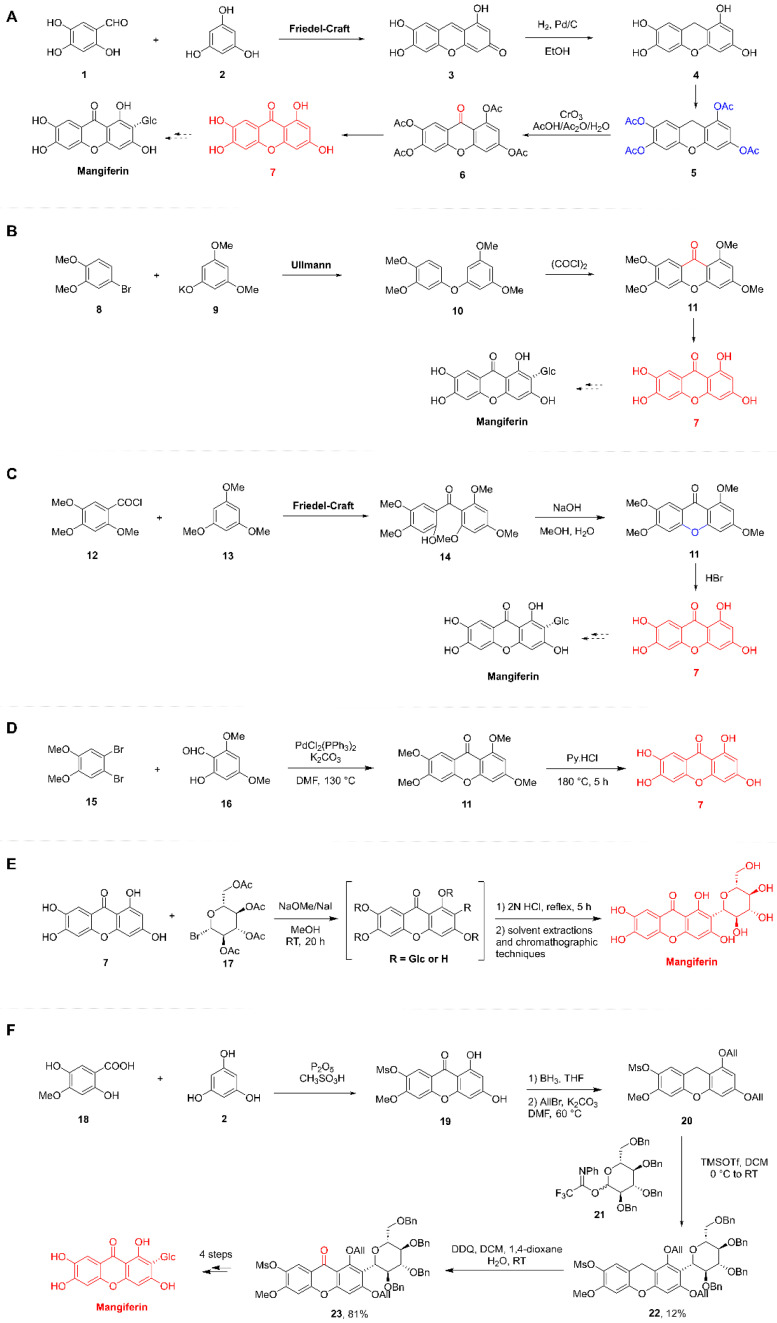
Total synthetic route of Mangiferin. (**A**,**B**): The Tanase research group conducted a study on the synthesis of tetrahydroxyxanthone (1941). (**C**): The Scheinmann research group reported the synthesis of tetrahydroxyxanthone (1973). (**D**): The Yang group developed an efficient synthesis of tetrahydroxyxanthone (2014). (**E**): The research team of Nott and Roberts achieved the synthesis of MF (1967). (**F**): The Yu group devised an efficient synthetic route to MF (2010).

**Figure 5:**
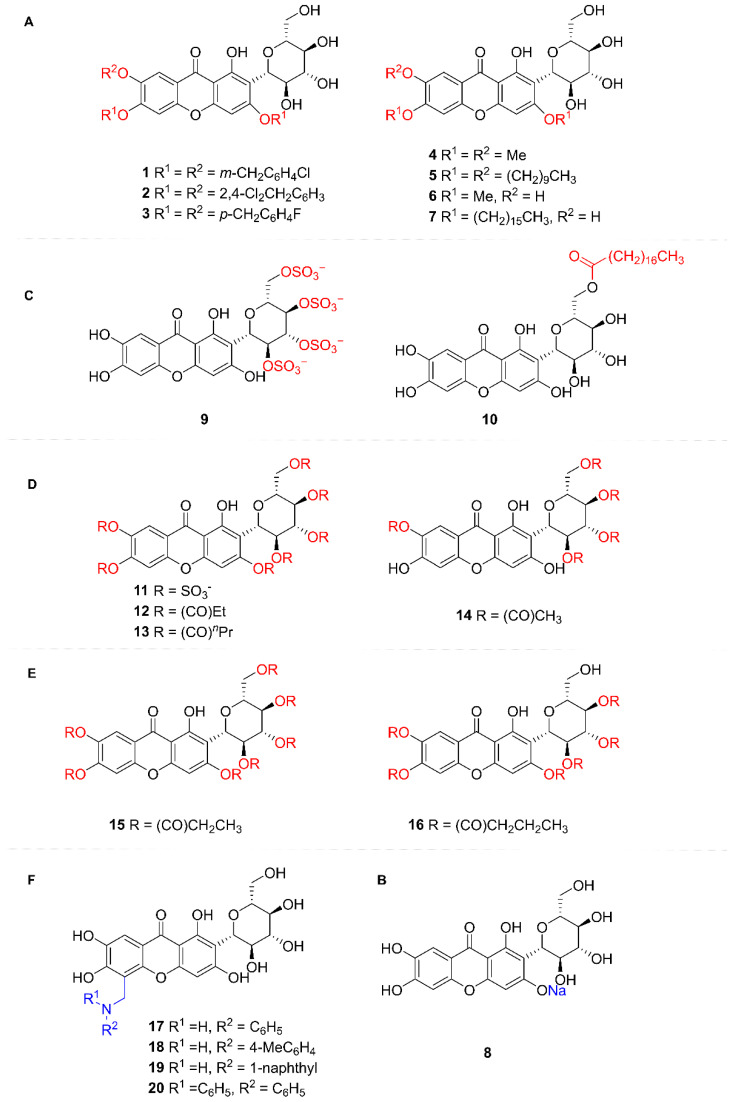
Structural Modification Schematic of Mangiferin. (**A**) Hydroxy groups on the xanthone nucleus; (**B**) Sodium phenolate salts on the xanthone nucleus; (**C**) Hydroxy groups of glucose; (**D**) Hydroxy groups of mangiferin; (**E**) Oxygenated xanthone core’s C-8 position; (**F**) Nitrogen-containing derivatives of mangiferin.

The chemical synthesis of xanthones has attracted considerable interest from organic chemists due to their inherent biological significance. Starting from the original substrates 2,4,5-trihydroxybenzaldehyde **(1)** and phloroglucinol **(2)**, compound **3** was obtained via a Friedel-Crafts reaction. Compound **4** was then obtained via hydrogenation, compound **5** was obtained via complete acetylation, compound **6** was obtained by oxidizing the C-9 position of compound **5** with CrO_3_, and it was hydrolyzed under acidic conditions to compound **7**, which is tetrahydroxyoxazolone (Figure 4A). Additionally, they also employed 4-bromoveratrole **(8)** and potassium 3,5-dimethoxyphenoxide **(9)** to obtain the coupling product **10** via an Ullmann reaction. Subsequently, compound **10** underwent a Friedel-Crafts reaction in the presence of oxalyl chloride to afford compound **11**. Demethylation of compound **11** then yielded compound **7** [37] (Figure 4B). Researcher synthesized tetrahydroxyxanthone using a Friedel-Crafts reaction, developed by Shah, as the key step. This involved the reaction of 2,4,5-trimethoxybenzoyl chloride **(12)** with 1,3,5-trimethoxybenzene **(13)** under ZnCl_2_-POCl_3_ conditions to afford the corresponding benzophenone intermediate **14** [47] (Figure 5C). Thus, 2,4,5-trimethoxybenzoyl chloride **(12)** and 1,3,5-trimethoxybenzene **(13)** were subjected to a Friedel-Crafts reaction in the presence of AlCl_3_ to yield compound **14**. Subsequent intramolecular cyclization of **14** in aqueous methanolic NaOH furnished compound **11**. Demethylation of **11** under refluxing HBr then afforded compound **7** (Figure 4C). Thus, 2,4,5-trimethoxybenzoyl chloride **(12)** and 1,3,5-trimethoxybenzene **(13)** were subjected to a Friedel-Crafts reaction in the presence of AlCl_3_ to yield compound **14**. Subsequent intramolecular cyclization of **14** in aqueous methanolic NaOH furnished compound **11**. Demethylation of **11** under refluxing HBr then afforded compound **7** [47,48] (Figure 4D).

This was followed by the stereoselective construction of the C-glycosidic bond. In traditional carbohydrate chemistry, the stereoselective formation of C-glycosidic bonds presents significantly greater challenges than that of O-glycosidic bonds. The total synthesis required implementation via either Friedel-Crafts glycosylation or Lewis acid-catalyzed C-glycosylation reactions. However, these reactions can be accompanied by the formation of byproducts and pose difficulties in stereochemical control.

This pioneering work established the C-glycosidic bond via Friedel-Crafts glycosylation, utilizing the electron-rich flavonoid nucleus (xanthone core) as the acceptor and glucose as the donor, culminating in the total synthesis of MF. Specifically, compound **7** was reacted with an excess of *α*-acetobromoglucose **(17)** in anhydrous methanol containing sodium methoxide/sodium iodide, yielding a mixture of C- and O-glucosylated xanthones. Acidic treatment with diluted hydrochloric acid selectively cleaved the O-glucosidic bond while leaving the C-glucosidic bond intact. Subsequent extraction and chromatographic separation finally afforded MF, albeit in a yield of only 0.1% (Figure 4E). Researchers have also conducted studies using such methods. However, the yield remained similarly low at merely 0.05%. Despite the extremely low yields, this strategy achieved the selective construction of the C-glycosidic bond, laying the essential groundwork for the subsequent optimization of the synthetic route [49] (Figure 4E).

Researchers have also conducted studies on the total synthesis of MF. Recognizing that the electron-withdrawing effect of the C-9 carbonyl group diminishes the nucleophilicity of the xanthone nucleus, researchers opted to perform the C-glycosylation on a xanthone precursor prior to introducing the carbonyl group at C-9. The synthesis commenced with the construction of the xanthone nucleus **(20)**, and achieved in three steps: (a) Condensation of 4-methoxy-2,5-dihydroxybenzoic acid **(18)** with phloroglucinol **(2)** using P_2_O_5_/CH_3_SO_3_H afforded xanthone **19**, (b) Reduction in the C-9 carbonyl group in **19** with BH_3_ yielded the corresponding xanthene intermediate, (c) Allylation of the 1,3-dihydroxy groups furnished the desired xanthone nucleus **20**. Subsequently, glycosylation of xanthone nucleus **20** with 2,3,4,6-tetra-O-benzyl-D-glucopyranosyl N-phenyl trifluoroacetimidate **(21)** under TMSOTf/DCM conditions afforded the target C-glycoside **22** in 12% yield. This was followed by oxidation of glycoside **22** with DDQ to introduce the carbonyl group at C-9, providing the benzylated xanthone glycoside **23** in 81% yield. Finally, sequential removal of the allyl, mesyl (methylsulfonyl), methyl, and benzyl protecting groups delivered MF. Notably, the allyl protecting group proved indispensable for successful glycosylation, as analogous attempts using benzyl or methoxy protecting groups under similar conditions failed to afford the desired glycoside [50] (Figure 4F).

#### 5.2.2. Structural Modification of MF

MF is a naturally occurring flavonoid C-glycoside. Its core structure consists of a chromone scaffold bearing a glucosyl group attached at the C-2 position via a C-glycosidic bond. Structural modifications of its derivatives are primarily focused on the hydroxy groups and the aglycone scaffold, aiming to optimize solubility, pharmacological activity, and targeting specificity.

(1) Hydroxy groups on the xanthone nucleus (Benzyl, alkyl, and other groups)

Studies revealed that introducing long-chain alkyl groups and bulky lipophilic substituents onto the MF core in compounds **4**–**7** significantly enhanced their inhibitory activity against protein tyrosine phosphatase 1B. Notably, compound **5** achieved complete (100%) inhibition of this enzyme at a concentration of 50 μmol/L [51] (Figure 5A).

(2) Sodium phenolate salts on the xanthone nucleus

Synthesis of MF monosodium salt was achieved under alkaline conditions by selective conversion of the 3-phenolic hydroxy group to its sodium phenolate salt. This modification significantly enhanced the aqueous solubility of the resulting product. Studies revealed that MF monosodium salt exhibits marked antitussive, expectorant, antiasthmatic, and anti-inflammatory activities. Notably, its antitussive, expectorant, and anti-inflammatory effects were significantly superior to those of MF. Subsequent research further demonstrated the ameliorative effects of MF sodium salt on alcoholic hepatitis [52,53,54,55] (Figure 5B).

(3) Hydroxy groups of glucose (Sulfonic group, acyl group)

MF was esterified with benzoic acid, stearic acid, and phenylalanine to yield amphiphilic MF ester derivatives. These derivatives exhibited significantly enhanced solubility compared to MF, facilitating improved cellular uptake and augmenting their antioxidant activity. They demonstrated superior efficacy in inhibiting adipocyte differentiation and lipid droplet formation, suppressing leptin, pro-inflammatory cytokines, and oxidative stress responses, while promoting adiponectin secretion. These properties suggest their potential to delay obesity onset and mitigate obesity-associated precocious puberty. Notably, the stearic acid-modified MF derivative 10 displayed the most potent effects, positioning it as a promising therapeutic candidate for ameliorating obesity and preventing precocious puberty [56] (Figure 5C).

(4) Hydroxy groups of MF (sulfonic acid and acyl group)

The polysulfation of hydroxyl groups in MF yielded heptasulfated MF. The key modification lies in the introduction of sulfate groups, which enhances molecular negative charge and target binding capacity. Studies demonstrate that sulfated MF derivatives exhibit significantly improved solubility, stability, and anticoagulant/antiplatelet activities compared to the parent compound. These findings provide a theoretical foundation for the development of novel antithrombotic agents [57] (Figure 5D).

(5) Oxygenated xanthone core’s C-8 position

The introduction of various aromatic amine groups significantly alters the physicochemical properties of MF derivatives. Incorporation of lipophilic substituents potentially enhances membrane permeability, thereby improving bioavailability. Experimental data indicates that these analogs exhibit superior antipyretic activity to MF at 200 mg/kg doses, with naphthyl- and bis-phenylamino-containing derivatives demonstrating the most pronounced effects. This enhanced efficacy is hypothesized to correlate with improved antioxidant capacity and inhibition of TNF-*α* synthesis. Structural modifications through strategic manipulation of molecular polarity and steric effects successfully balance solubility characteristics with target engagement efficiency, offering a novel paradigm for developing highly efficient, low-toxicity antipyretic agents [58,59] (Figure 5E).

## 6. Pharmacokinetics of MF

**Figure 6:**
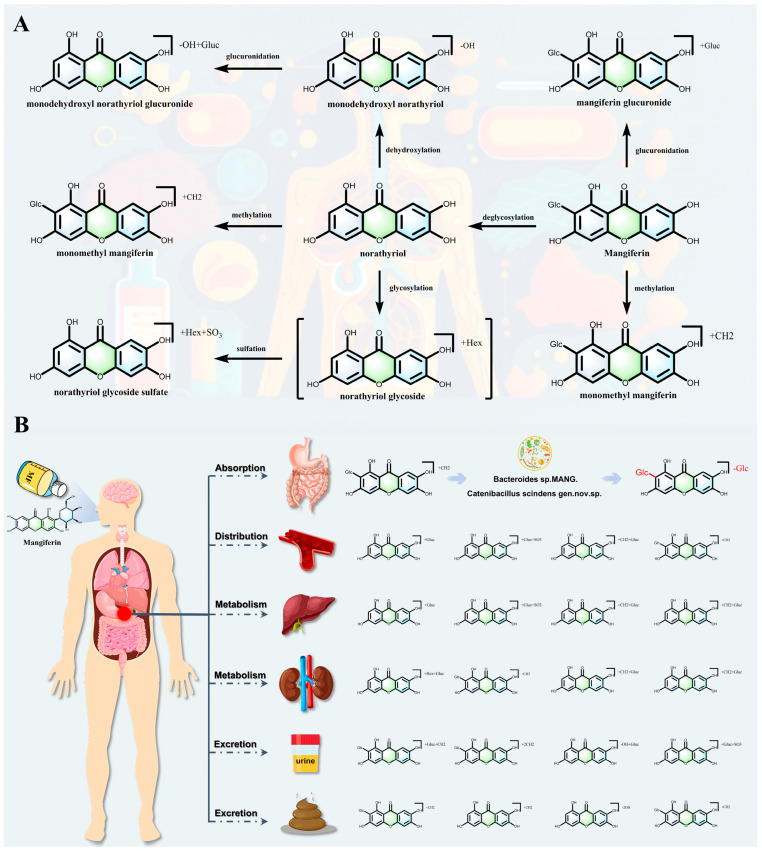
(**A**,**B**): The process of absorption, distribution, metabolism and excretion pattern of Mangiferin.

The pharmacokinetic process of MF involves a multi-stage biotransformation via multiple reactions, including glycosylation, deglycosylation, methylation, glucuronidation, sulfation, and dehydroxylation, and the intestinal flora plays a key regulatory role in the early stages of metabolism [60,61] (Figure 6A). This process can be divided into three sequential phases: initial activation by gut microbiota, systemic modification in the liver, and final excretion with potential recirculation.

In the intestinal flora-mediated metabolism, MF (C-glycoside) is specifically hydrolyzed by C-glycosidase secreted by intestinal commensal bacteria to produce the aglycone norathyriol. Additionally, some bacterial strains further catalyze the removal of secondary glycosidic bonds via *β*-glucosidase, resulting in low-glycosylated products [21,62,63,64]. This initial deglycosylation process is accompanied by in situ methylation, and colony metabolizing enzymes catalyze the methylation of phenolic hydroxyl groups to generate mono-or di-methylated derivatives, which significantly enhances the lipid-solubility of the metabolite and the efficiency of transmembrane absorption [65,66]. This microbial preprocessing is a crucial step that enables the efficient absorption of MF-derived metabolites.

Upon entry into the systemic circulation, the liver systematically regulates metabolite properties through phase II conjugation reactions: uridine diphosphate glucuronosyltransferases mediate glucuronidation, sulfotransferases catalyze sulfation, and glycosyltransferases may reintroduce secondary sugar groups to form novel O-glycoside derivatives. Furthermore, cytochrome P450 enzymes regulate the strength and targeting of activity by dehydroxylating to produce low-hydroxyl metabolites. In the excretion and recirculation chain, the resulting conjugates are excreted via bile or urine, and some of them can be hydrolyzed anew by intestinal flora to form an enterohepatic cycle, thereby prolonging their systemic exposure. Metabolomics studies based on UHPLC-Q-Exactive orbitrap HRMS have identified a variety of metabolites, revealing the complexity of the intestinal flora-hepatic synergistic metabolic network, among which the competitive relationship between methylation and glucuronidation, and the discovery of new metabolites of trihydroxyflavonoids provide a key basis for the optimization of the design of prodrugs and the evaluation of individualized therapeutic efficacy (Figure 6B). In conclusion, the metabolic kinetics of MF covers deglycosylation, methylation, glucuronidation, sulfation, glycosylation and dehydroxylation pathways, and its multi-organ synergistic mechanism not only elucidates the causes of low bioavailability, but also lays a theoretical foundation for the development of targeted metabolites [67,68,69].

**Figure 7:**
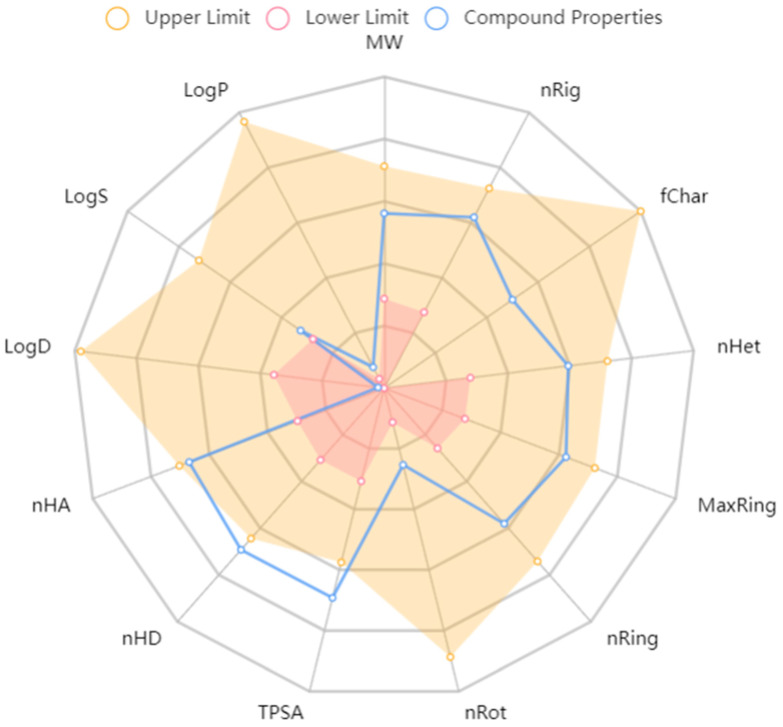
Radar chart involved in ADMET properties of mangiferin and relevant metabolites compared with the thresholds. Het, heteroatoms; fChar, formal charge; Rot, rotatable bonds; HD, hydrogen bond donors; MaxRing, atoms in the biggest ring; TPSA, topological polar surface area; LogS, solubility.

Complementing the experimental findings, in silico predictions provide a theoretical perspective on MF’s ADME properties. The ADME process of MF was predicted by the ADMETLab 3.0 platform and Deep ToxLab platform (Figure 7). According to the ADME prediction results, MF exhibits poor oral absorption (low bioavailability), difficulty in penetrating cell membranes and the blood–brain barrier, and limited distribution in the body due to its large molecular weight (approximately 422 g/mol), strong hydrophilicity (extremely low LogP/LogD), high polar surface area, and abundant hydroxyl groups (multiple hydrogen bond donors). Additionally, its polyphenolic hydroxyl structure is prone to phase II metabolism, leading to rapid metabolism and primarily excretion via the kidneys and bile, which aligns with the extensive conjugation observed experimentally.

## 7. Safety of MF

**Figure 8:**
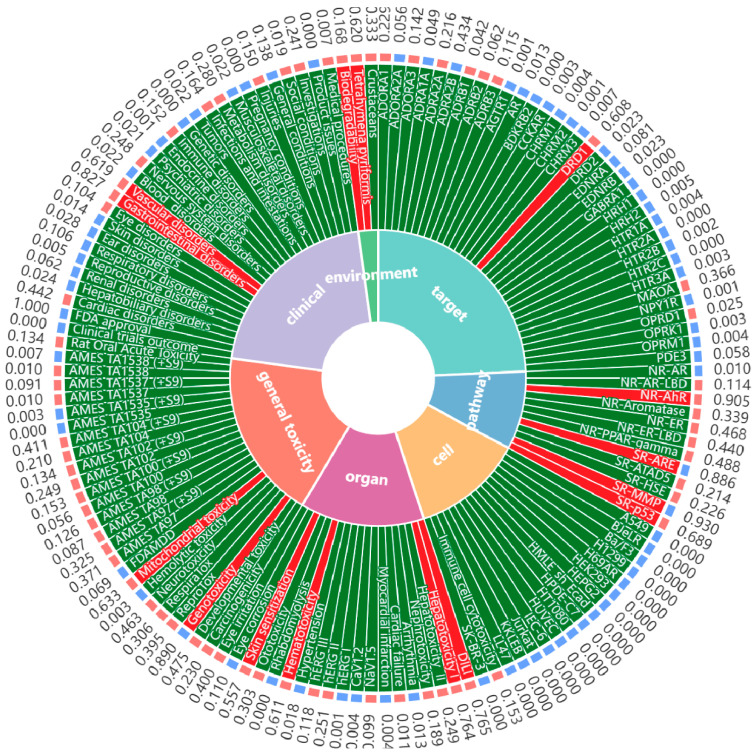
Toxicity prediction of mangiferin via Deep ToxLab.

The toxicology of MF was predicted by Deep ToxLab platform (Figure 8). Toxicological predictive analysis indicates that MF exhibits no significant toxicity risks across seven dimensions: target toxicity, pathway toxicity, cellular toxicity, organ toxicity, animal toxicity, clinical toxicity, and environmental toxicity, demonstrating good safety characteristics. This computational prediction is strongly supported by evidence from pre-clinical in vivo studies. Acute toxicity assessments have established that MF is practically non-toxic following oral administration, with a median lethal dose (LD_50_) reported to be as high as 4984 mg/kg body weight in mice. In sub-chronic toxicity studies, oral administration of MF at doses ranging from 250 to 1000 mg/kg for 28 days did not induce any abnormal clinical signs or hematological alterations in rats, indicating a wide safety margin for medium-term use [41]. These results further highlight MF’s favorable safety profile as a natural antioxidant, reinforcing its potential for therapeutic use with minimal toxicological concerns, thus enhancing its appeal as a viable candidate for clinical application.

## 8. Mechanism of MF in the Treatment of Liver Disease

According to the World Health Organization, about 20% of the global disease burden is related to liver disease. As the core organ of human metabolism and detoxification, the dynamic evolution of pathological injury in the liver is often characterized by multiple stages, covering acute liver injury, non-alcoholic fatty liver disease (including simple fatty liver and steatohepatitis), liver fibrosis, cirrhosis, and up to the malignant transformation of hepatocellular carcinoma [70,71,72]. In this pathological continuum, molecular mechanisms such as oxidative stress, mitochondrial dysfunction, inflammatory cascade and abnormal deposition of extracellular matrix are intertwined to drive the disease towards end-stage. Although some progress has been made in clinical intervention strategies, their efficacy is mostly limited to a single pathology, and there are problems such as low bioavailability, insufficient targeting, and toxicity of long-term drug use [73,74].

In recent years, natural product multi-target intervention strategies have attracted much attention due to both pharmacological activity and safety advantages. MF, a C-glucosylxanthones isolated from *Mangifera indica*, has been shown to be effective in regulating the nuclear factor E2-related factor 2 (Nrf2)/antioxidant response element pathway, inhibiting the activation of nucleotide-binding oligomerization structural domain-like receptor protein 3 (NLRP3) inflammatory vesicles, and antagonizing the activation of transforming factor-*β*. Growth factor-*β*/Smad signaling and other key mechanisms have shown multidimensional regulatory potential, providing an important direction for the development of novel therapeutic strategies for liver disease [75,76].

In the field of liver disease research, there are various modeling methods for different stages of liver disease, and MF has demonstrated unique mechanisms and pharmacological activities in the treatment of various stages of liver disease [77].

### 8.1. Acute Liver Injury

**Figure 9:**
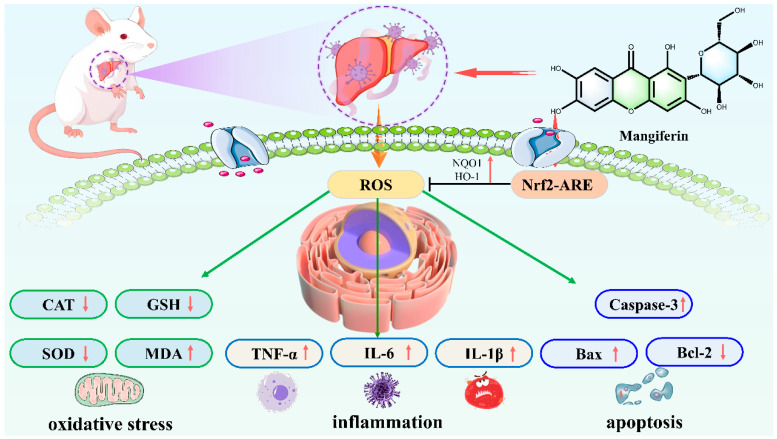
Mangiferin against Polar Liver Injury mechanism diagram.

In acute liver injury, lipopolysaccharide combined with D-galactosamine, acetaminophen, and carbon tetrachloride (CCl_4_) are often used to induce modeling. The mechanism of MF treatment of acute liver injury mainly involves the regulation of multiple signaling pathways and intracellular processes, which promotes the expression of antioxidant enzymes and enhances the body’s antioxidant capacity to inhibit oxidative stress injury by activating the Nrf2 pathway, inhibits the activation of NLRP3 inflammatory vesicles, and regulates the endoplasmic reticulum stress-related miR-20a/miR-101a-Nrf2 axis to maintain endoplasmic reticulum homeostasis [78]. It can also promote the expression of heme oxygenase-1 (HO-1) in Kupffer cells, inhibit the production of inflammatory factors such as TNF-*α*, and exert hepatoprotective effects. Its pharmacological activity is manifested in significantly reducing the serum levels of liver injury markers such as alanine aminotransferase and azelaic transaminase and alleviating pathological changes such as hepatocyte degeneration and necrosis [79] (Figure 9).

### 8.2. Fatty Liver

For fatty liver, a model is usually constructed using high-fat-diet-fed animals. MF can regulate fat metabolism-related signaling pathways, activate the AMPK pathway, inhibit the activity of fatty acid synthase and other key enzymes of lipid synthesis, promote fatty acid oxidation, and reduce hepatic fat accumulation [80]. Meanwhile, it can inhibit inflammatory responses, reduce the expression of inflammatory factors such as TNF-*α* and IL-6, and improve the hepatic inflammatory microenvironment, thus alleviating fatty liver symptoms.

By improving lipid metabolism through AMPK activation and inhibition of fatty acid synthase, MF alleviates hepatic lipotoxicity and steatosis, thereby establishing a metabolic link between its systemic lipid-regulatory actions and liver protection.

### 8.3. Steatohepatitis

The modeling method for steatohepatitis is often alcohol gavage combined with high-fat diet-treated animals [81,82]. MF not only regulates lipid metabolism and reduces the accumulation of triglycerides and free fatty acids in the liver at this stage, but also reduces the level of inflammatory factors by inhibiting the inflammatory signaling pathway, such as NF-κB, to reduce the inflammatory injury of hepatocytes and to improve liver function [83].

### 8.4. Liver Fibrosis

Hepatic fibrosis models are mostly induced by repeated intraperitoneal injections of CCl_4_. The mechanism of hepatic fibrosis treatment with MF is more complex. On the one hand, it inhibits the activation and proliferation of hepatic stellate cells by inhibiting the TGF-*β*1/Smad pathway and reducing the synthesis and deposition of extracellular matrix. On the other hand, MF can target the HSP27-mediated JAK2/STAT3 and TGF-*β*1/Smad pathways, preventing the hepatocyte epithelial–mesenchymal transition, maintaining the normal morphology and function of hepatocytes [84,85]. In addition, MF can activate Nrf2, protect DNase 2 abundance, prevent inflammatory reactions triggered by cytoplasmic mtDNA accumulation, and thus reduce the degree of liver fibrosis [86].

### 8.5. Liver Cirrhosis

Cirrhosis modeling can be done by long-term induction of CCl_4_ and bile duct ligation combined with infection [87,88]. MF plays a therapeutic role in cirrhosis by regulating the metabolic function of the liver, improving the microcirculation of hepatic tissues, promoting the regeneration of hepatocytes, and inhibiting the process of inflammation and fibrosis.

### 8.6. Liver Cancer

In liver cancer research, diethylnitrosamine is commonly used to induce rat liver cancer model. MF has the effect of inhibiting the proliferation of hepatocellular carcinoma cells and inducing apoptosis, which can regulate cell cycle-related proteins and block the progression of the cancer cell cycle. Meanwhile, it inhibits the migration and invasive ability of cancer cells and down-regulates the expression of related proteins, such as MMP-9. It can also alleviate the damage of hepatocellular carcinoma cells on the liver by altering the pathways of oxidative stress and apoptosis [87,89].

**Figure 10:**
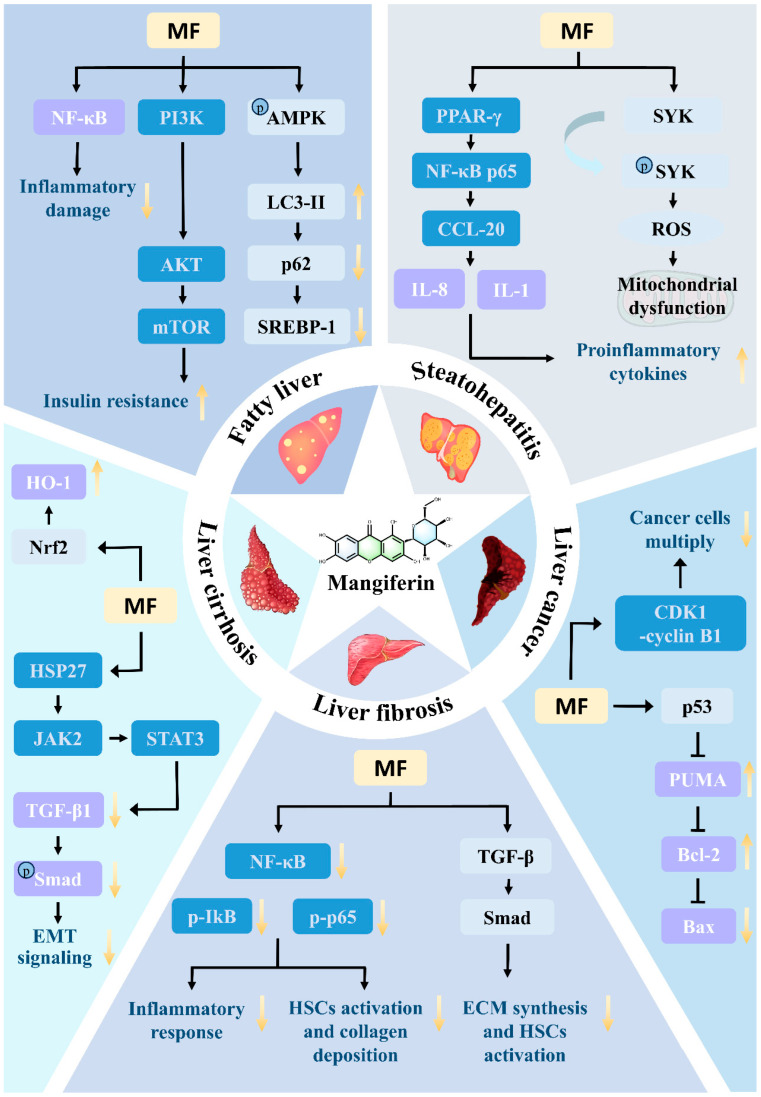
Mechanism of mangiferin in treating liver diseases.

In summary, MF has demonstrated multi-pathway and multi-target therapeutic potentials in all stages of liver disease, providing a new direction and hope for the treatment of liver disease, but most of the studies are still in the stage of animal experiments, and its wide application in the clinic requires further in-depth research and validation (Table 2 and Figure 10).

## 9. Network Pharmacology Analysis of MF

### 9.1. Collection Targets of Liver Disease Genes

**Figure 11:**
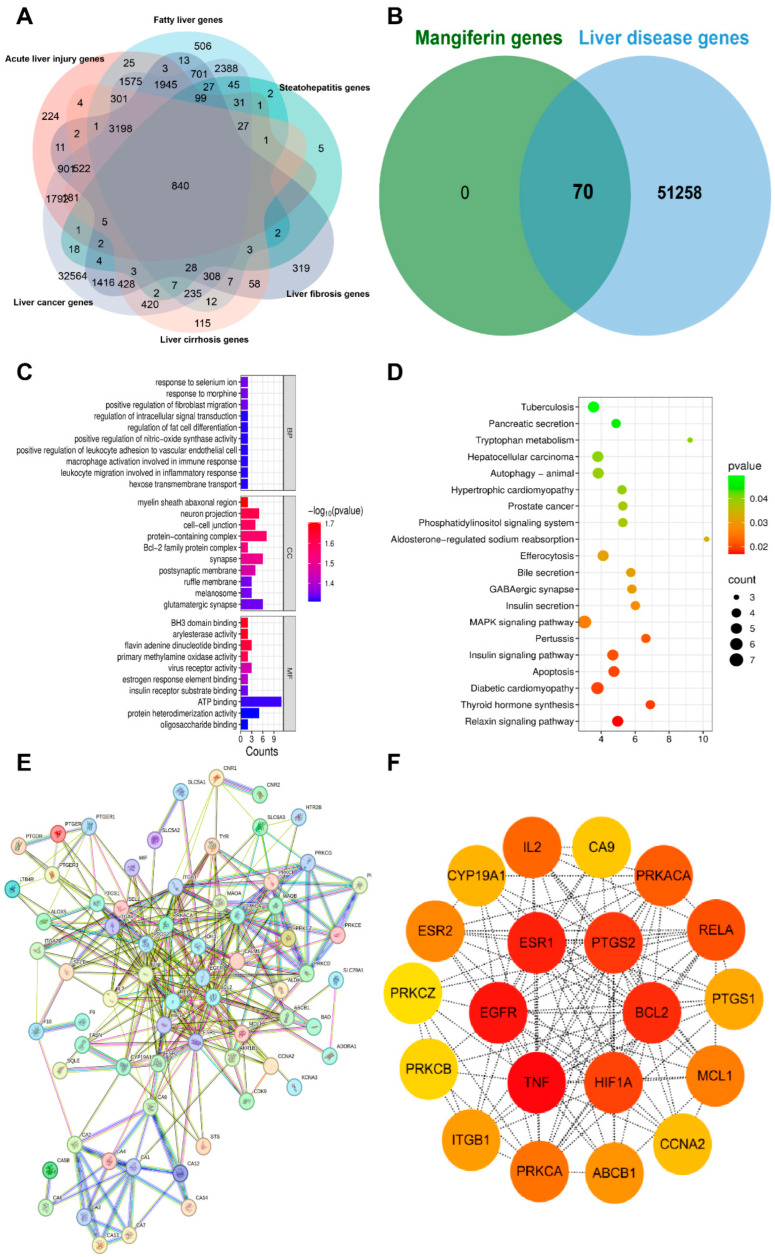
(**A**). Venn diagram of liver diseases. (**B**). Venn diagram of liver disease targets and mangiferin therapeutic targets. (**C**,**D**). KEGG and GO Enrichment Analysis Plots (**E**,**F**). Protein–protein interaction in network of Targets.

By using the online platform Draw Venn Diagram to find the intersection between the screened ingredient targets and disease targets, and plotting a Venn diagram, we obtained 70 targets for the therapeutic effect of MF in liver disease genes (Figure 11A,B).

### 9.2. GO and KEGG Pathway Enrichment Analysis

The findings from the enrichment analysis revealed a total of 183 BPs, 46 CCs, 49 MFs, and 112 KEGG pathways, with a significance level of *p* < 0.01. The illustration presents enriched GO, KEGG categories and component-target-pathway. Molecular functions encompass various activities such as oligosaccharide binding, protein heterodimerization activity, and ATP binding, among others. The cellular components identified glutamatergic synapse, ruffle membrane, and melanosome. Additionally, the biological processes involve hexose transmembrane transport, positive regulation of leukocyte adhesion to vascular endothelial cell, and macrophage activation involved in immune response, among other functions. The twenty most prevalent KEGG pathways are presented, which include pathways related to tuberculosis, pancreatic secretion, tryptophan metabolism, hepatocellular carcinoma and autophagy-animal, among others (Figure 11C,D).

### 9.3. Protein–Protein Interaction (PPI) Network Construction

The potential targets of MF for the treatment of liver diseases were imported into the STRING database to obtain the PPI network, and the topological analysis showed that the PPI network contained 70 nodes and 301 edges. The data were imported into Cytoscape 3.8.0 software, and the topological analysis was performed by using CytoNCA plug-in in Cytoscape 3.8.0 software, and 20 core targets were screened by using the median of the degree value as the standard, including ESR1, PTGS2, BCL2, HIF1A, TNF, EGFR, IL2, PRKACA, RELA, PRKCA and other core targets, of which the top six targets were TNF, EGFR, ESR1, BCL2, PTGS2 and HIF1A (Figure 11E,F).

### 9.4. Molecular Docking Analysis

**Figure 12:**
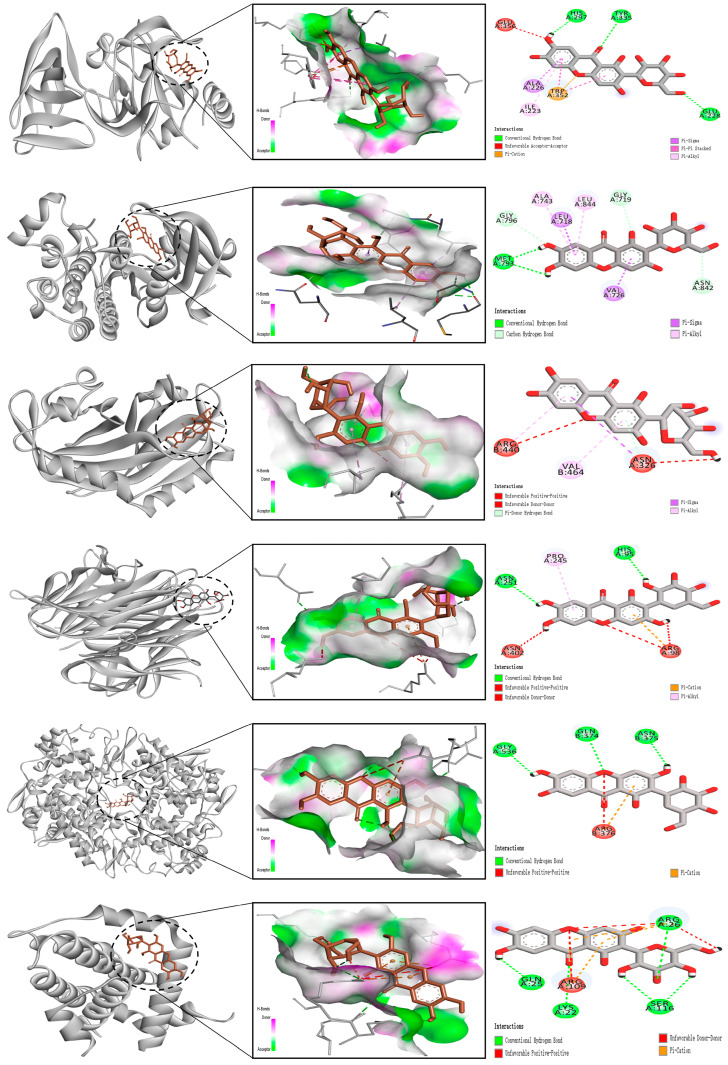
Molecular docking diagram of mangiferin with key liver disease targets (ESR1, PTGS2, BCL2, HIF1A, TNF, EGFR).

The receptor proteins were ESR1 (3cbp), PTGS2 (3poz), BCL2 (4h6j), HIF1A (4zch), TNF (5f19), and EGFR (8hts), and their 3D structure files were downloaded from the PDB database. PyMOL 2.3.0 software was used to check the protein structure for docking. The ligand small molecule is MF. Employ Auto Dock Vina (version 1.1.2) to perform docking of the compounds with the target. MF forms hydrogen bonds with the HIS, TYR, GLU, TRP, ILE, and ALA amino acid residues in ESR1, with bond lengths of 297, 335, 228, 352, 223, 226, and 356. MF forms hydrogen bonds with the ALA, LEU, GLY, ASN, VAL, and MET amino acid residues in PTGS2, with bond lengths of 743, 718, 844, 719, 842, 726, 793, and 796. MF forms hydrogen bonds with the ARG, VAL, and ASN amino acid residues in BCL2, with bond lengths of 440, 464, and 326. MF forms hydrogen bonds with the ASN, PRO, HIS, and ARG amino acid residues in HIF1A, with bond lengths of 251, 245, 95, 98, and 402. MF forms hydrogen bonds with the GLY, GLN, ASN, and ARG amino acid residues in TNF, with bond lengths of 536, 374, 375, and 376. MF forms hydrogen bonds with the ARG, SER, LYS, ARG, and GLN amino acid residues in EGFR, with bond lengths of 26, 116, 22, 109, and 25 (Figure 12).

## 10. Study on the Bioavailability Enhancement Strategy of MF and Its Targeted Therapeutic Mechanism

MF exhibits limited solubility in water, which significantly contributes to its low oral bioavailability. Specifically, MF is described as being sparingly soluble in water (as well as in methanol), while it is insoluble in non-polar solvents such as diethyl ether, acetone, and n-hexane. This poor aqueous solubility, combined with its stability as a C-glycoside that resists hydrolysis in the gastrointestinal tract, limits its absorption in the small intestine. As a result, when MF is consumed orally, only a small fraction is absorbed via passive diffusion, leading to low systemic bioavailability. To address this issue, various strategies such as structural modification, nano/micro-encapsulation, and the use of solubility-enhancing carriers have been explored to improve its water solubility and overall bioavailability [41].

Due to the poor water solubility of MF, its bioavailability in the human body is also significantly limited. The oral bioavailability of MF in humans is very low, which is a major limitation for its efficacy. In a clinical study involving healthy male Chinese volunteers, a single high oral dose of 0.9 g of MF resulted in a maximum plasma concentration (Cmax) of only 38.64 ng/mL, which was reached after about 1 h (Tmax). The mean residence time (MRT) and elimination half-time (t_1/2_) were 13.87 h and 7.85 h, respectively. The very low plasma concentration achieved from a high dose clearly demonstrates the poor systemic availability of MF in humans [41].

This low bioavailability is attributed to its poor solubility, passive diffusion-based absorption in the intestine, and significant hepatic first-pass metabolism. Consequently, the biological effects observed after oral intake are likely due to its metabolites (such as norathyriol) produced by the gut microbiota, which have higher bioavailability, rather than the parent MF molecule itself.

### 10.1. New Dosage Form Development: Multimodal Carrier Systems Reshape Drug Delivery Characteristics

**Figure 13:**
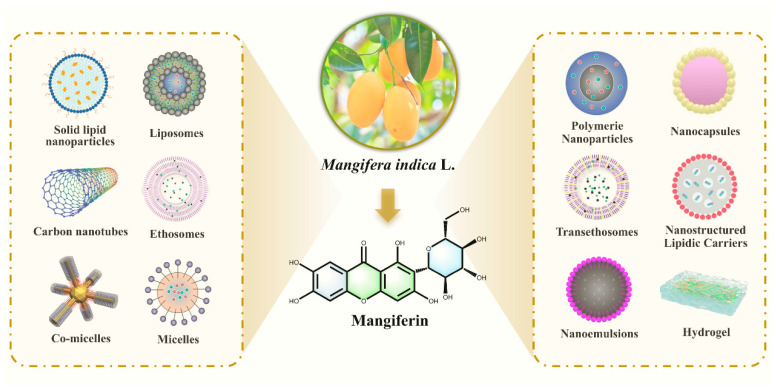
Advanced delivery systems for mangiferin.

The new dosage form development systematically improves the solubility, membrane permeability and in vivo distribution characteristics of MF through the synergistic design of nanotechnology, colloidal carriers and extended release systems [90,91,92,93]. Nanoparticles based on amphiphilic polymers are formed into a core–shell structure by the method of complex emulsion-solvent evaporation, encapsulating the drug in a hydrophobic core, with an outer hydrophilic coating avoiding the body’s elimination, which improves the solubility by 1.79-fold, the oil-water partition coefficient by 2.04-fold, the rate of dissolution by 1.48-fold compared with that of active pharmaceutical ingredients (API), and significantly extends the in vivo residence time, with a relative bioavailability of 162.0-fold [94,95,96]. Solid dispersion technology utilizes water-soluble carriers to highly disperse the drug in an amorphous state, destroying the crystalline structure, increasing solubility by a maximum of 633 times, and increasing the cumulative release rate from 30.5% to 76.1% within 5 min. It can also reduce the contact of intestinal bacteria and slow down the metabolic degradation through the hydrogen bonding effect between carrier molecules [97,98,99]. Lipid-based carriers solubilize hydrophobic drugs with the help of phospholipid bilayers or self-assembly properties to optimize the transmembrane permeation efficiency, among which, the self-microemulsion drug delivery system increases the peak concentration by 7.7-fold and shortens the time to peak to 0.358 h, which significantly improves the oral absorption kinetics [100,101,102,103]. In addition, the porous microspheres and mesoporous silica nanoparticles control the drug release through the three-dimensional pore structure and delay the intestinal degradation. The residual concentration of the drug at 4 h is more than three times that of the raw material drug, which provides the carrier basis for the long-lasting delivery [104,105] (Figure 13).

### 10.2. Structural Modification: Target Potentiation and Hepatic Injury Therapeutic Mechanism of MF Sodium Salt

**Figure 14:**
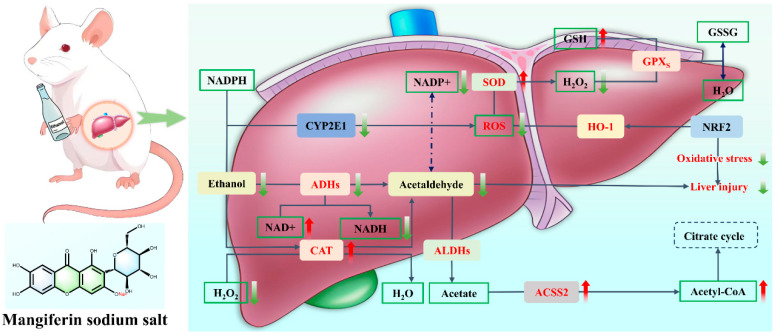
Molecular mechanism of mangiferin sodium salt against alcoholic fatty liver disease.

The structural modification optimizes the physicochemical properties of MF through chemical modification, in which the sodium salt form significantly enhances the water solubility through phenolic hydroxyl salt formation, increasing the solubility from 0.123 mg/mL to more than 1.2 mg/mL, and at the same time regulates the molecular polarity to promote transmembrane transport and target enrichment in liver tissues. In the treatment of alcoholic liver injury, MF sodium salt can regulate the activity of key enzymes of alcohol metabolism, inhibit the over-activation of ethanol dehydrogenase and the accumulation of acetaldehyde, reduce the oxidative stress damage in the liver, lower the level of serum alanine aminotransferase and aspartate aminotransferase. Meanwhile, it enhances the antioxidant reserve of glutathione, inhibits the expression of inflammatory factors, and alleviates the lipid peroxidation of the hepatic tissues and the deposition of collagen fibers [106]. At the pharmacokinetic level, the negative charge property of sodium salt enhances the binding with anion receptor of liver cell membrane, so that the drug concentration in liver tissue reaches the peak within 1 h, which is 2.4 times higher than that of API; the plasma protein binding rate is increased from 65% to 82%, the circulation time is prolonged, the first-pass metabolism is reduced, and the clearance rate is reduced by 1.61 times, which significantly widens the therapeutic window. Mechanistic studies showed that the modified form promoted hepatocyte repair by regulating the glycerophospholipid metabolic pathway, inhibiting pro-inflammatory signaling, and reducing the degree of fibrosis by 50% in the CCl_4_-induced chronic liver injury model, demonstrating the advantages of structural modification in enhancing bioavailability while targeting and protecting against liver injury [55] (Figure 14).

### 10.3. Biotransformation: Bacterial Colony Intervention and Metabolic Pathway Modulation to Enhance Drug Stability

At the biotransformation level, the C-glucosidic bond of MF is easily hydrolyzed by intestinal anaerobic flora, resulting in a significant first-pass effect, so the dosage form design can intervene in this process through physical barriers and biochemical modulation [107]. The amorphous dispersion and spatial site-blocking effect of solid dispersions and nanoparticles reduce the contact area between the drug and glycosidase, which reduces the metabolism rate by 40%. The concentration of unmetabolized drug in 4 h is 2 times higher than that of API, which prolongs the intestinal retention time. Phospholipid complex with surface-modified nanoparticles enhances the transmembrane transport efficiency by 1.8-fold by inhibiting the activity of intestinal P-glycoprotein efflux pumps, reducing the reverse drug excretion. The slow-release properties of microspheres and lipid carriers control the release rate of the drug, so that the metabolite norcoronarin is continuously generated, exerting synergistic antioxidant and anti-inflammatory effects. The concentration of active metabolites in liver tissue is increased 3-fold compared with that of the free drug, thus prolonging the duration of action. In addition, the carrier system indirectly protects the structural integrity of the drug by targeting and regulating the composition of the intestinal flora and inhibiting the expression of degradative enzymes in dominant bacteria such as *Mycobacterium* spp. The proportion of the prototypic drug entering the circulation is increased by 35%, which circumvents the first-pass metabolism depletion and enhances the bioavailability. By intervening in the key link of biotransformation, this strategy significantly enhances the stability of the drug in the gastrointestinal tract and liver without changing its molecular structure, providing an optimized pathway at the metabolic level for the development of oral formulations.

## 11. Future Perspectives

MF, a nutritional supplement widely distributed in various fruits, vegetables, and herbs, has garnered considerable attention for its remarkable antioxidative properties and therapeutic potential. As a potent bioactive compound, MF plays a crucial role in alleviating oxidative stress, a key contributor to the pathogenesis of many diseases, including liver disorders. Its strong antioxidant capabilities not only protect against oxidative damage but also promote liver health by mitigating lipid peroxidation and modulating inflammatory pathways.

While MF shows promise in preventing and treating liver diseases such as fatty liver and fibrosis, the therapeutic applications of MF are still constrained by its low bioavailability and poor solubility. Current strategies focus on enhancing its pharmacokinetic profile through structural modifications and novel delivery systems, which will facilitate its clinical application.

**Figure 15:**
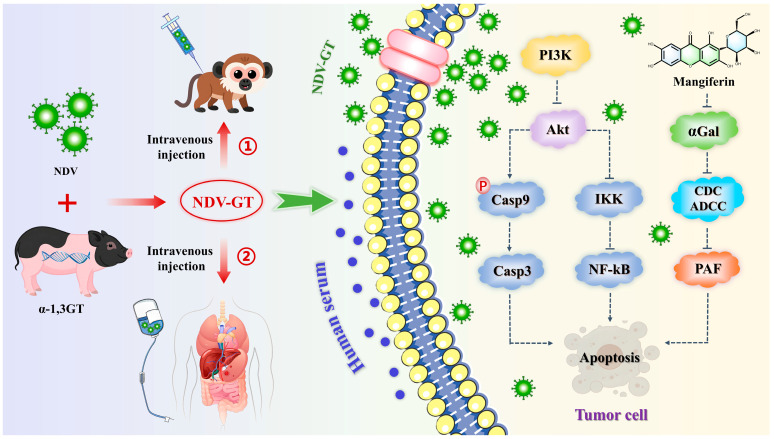
Molecular mechanism of NDV-GT and mangiferin in the treatment of hepatocellular carcinoma (*α*Gal: alpha-galactosidase, CDC: complement-mediated cytolysis, ADCC: antibody-dependent cell-mediated cytolysis, PAF: platelet-activating factor).

Hepatocellular carcinoma, a highly prevalent and poorly prognostic malignant tumor worldwide, remains primarily treated through surgical resection, liver transplantation, and systemic therapy. Liver transplantation is a critical means for advanced Hepatocellular carcinoma patients to achieve long-term survival. However, organ donor shortages, postoperative immune rejection reactions, and the risk of tumor recurrence significantly limit its widespread application [108,109,110]. Xenotransplantation offers a potential solution to the donor shortage, but the *α*1,3-galactosyl epitope triggers an ultra-acute rejection reaction mediated by the recipient’s natural anti-*α*1,3-galactosyl antibodies, leading to rapid graft necrosis [111]. To address these challenges, researchers have inserted the pig-derived *α*1,3-galactosyl transferase gene into Newcastle disease virus (NDV) to construct a recombinant oncolytic virus, Newcastle disease virus-pig-derived *α*1,3-galactosyl transferase gene. This virus selectively infects tumor cells and expresses the *α*1,3-galactosyl epitope, activating antibody-dependent cellular cytotoxicity mediated by anti-*α*1,3-galactosyl epitope antibodies, antibody-dependent cytolytic activity, and platelet-activating factor-induced tumor vascular thrombosis, as well as synergistically promoting CD4^+^/CD8^+^ T cell infiltration and other immune cascade reactions. This approach induced tumor regression in a CRISPR-mediated cynomolgus monkey hepatocellular carcinoma model and achieved a disease control rate of 90.00% in a clinical study involving 20 patients with refractory cancers, including hepatocellular carcinoma, with no severe adverse reactions. This technology leverages the immune mechanisms of hyperacute rejection, offering a novel approach for the clinical translation of oncolytic virus therapy and the regulation of xenograft rejection [112,113] (Figure 15). Future research may focus on optimizing viral vector design, exploring combination therapy with immune checkpoint inhibitors, and expanding indications. Further validation of long-term safety and immune memory effects is needed. Based on this, MF is increasingly being recognized for its potential role in regulating allograft rejection reactions. Studies have shown that MF can mitigate immune rejection reactions by inhibiting T cell activation, downregulating the secretion of pro-inflammatory cytokines, and blocking the activation of the classical complement pathway. In animal models, MF significantly reduces the infiltration levels of CD4^+^/CD8^+^ T cells in acute rejection reactions and inhibits antigen presentation by regulating the maturity of dendritic cells. For the hyperacute rejection response specific to xenotransplantation, MF may delay thrombosis formation in graft vessels by inhibiting the production of anti-*α*1,3-galactoside antibodies and the release of complement components C3a and C5a (Figure 15). However, current studies are primarily limited to in vitro experiments and rodent animal models [114,115,116]. The efficacy and pharmacokinetic characteristics of MF in non-human primate xenotransplantation models require further validation, particularly regarding its inhibitory mechanism against complement-dependent cytotoxicity mediated by natural antibodies and synergistic strategies when combined with immunosuppressive agents. These areas will become key research directions for overcoming the immune barriers in xenotransplantation.

Under the backdrop of the deep integration of precision medicine and green pharmaceutical concepts, research on MF for liver disease treatment urgently needs to break away from traditional perspectives and expand into multidimensional interdisciplinary fields. Regarding the regulation mechanism of the gut-liver axis, given the central role of the gut microbiota-bile acid metabolism axis in the onset and progression of liver diseases, it is imperative to elucidate the molecular target network through which MF regulates the gut barrier function, inhibits the colonization of pathogenic bacteria, and regulates short-chain fatty acid and secondary bile acid metabolic pathways, particularly its bidirectional regulatory mechanism on the FXR/TGR5 signaling pathway and intestinal immune cells. This may reveal the new pharmacological basis for MF’s “gut-liver co-treatment” effect [117,118]. As a natural flavonoid glycoside compound, MF demonstrates potential anti-inflammatory, antioxidant, and anti-fibrotic effects in liver disease treatment. However, its limited water solubility and targeting capacity hinder clinical application. Based on structural modification research progress, introducing specific functional groups can significantly optimize its pharmacological properties. Hydrophilic groups: Drawing on the structural modification experience of sodium myricetin sulfate, introducing sulfonic acid groups or glycosylation modifications to the phenolic hydroxyl group of mango glycoside can improve its water solubility, promote liver-targeted delivery, and enhance regulation of lipid metabolism disorders in non-alcoholic fatty liver disease. Nitrogen-containing heterocycles: Similarly to the design of piperazine ring derivatives in the patent, such groups may enhance the binding affinity between the molecule and liver cell surface receptors, thereby improving the inhibitory efficiency of liver fibrosis-related pathways while enhancing transmembrane permeability. Alkoxy or halogen substituents: Introducing methoxy or fluorine atoms at specific positions on the thioxanthone skeleton can regulate molecular hydrophobicity and enhance interactions with inflammatory signaling pathways such as NF-κB, thereby inhibiting hepatic stellate cell activation and delaying the progression of liver fibrosis. In the field of microbial heterologous synthesis pathways, cutting-edge synthetic biology technologies can be leveraged to reconstruct the metabolic pathways of chassis cells via CRISPR-Cas9-mediated editing, overcoming the carbon flux allocation limitations of natural MF synthesis. Combined with directed evolution techniques to optimize the catalytic efficiency of key enzymes, this approach enables the construction of an efficient whole-cell biosynthetic system, providing a green manufacturing solution to address resource acquisition bottlenecks. The application prospects of organ-on-a-chip technology lie in constructing a 3D liver micro-organism model containing hepatic sinusoidal endothelial cells, hepatic stellate cells, and Kupffer cells based on microfluidic chips, combined with an intestinal epithelial chip to build a liver-intestine axis serial system. This can precisely simulate the in vivo pharmacokinetic characteristics of MF and the cascade reaction of intestinal toxins causing liver damage, particularly suitable for assessing its dynamic intervention effects on myofibroblast activation and extracellular matrix remodeling during the liver fibrosis process [119,120,121,122].

## 12. Conclusions

In conclusion, MF stands out as a promising natural antioxidant with significant therapeutic potential for liver diseases. Its antioxidant properties, coupled with ongoing research into its bioavailability and delivery mechanisms, will likely lead to the development of innovative therapies that can effectively address liver disease and beyond, demonstrating both scientific and translational value.

## Figures and Tables

**Table 2:** The pharmacological effects and mechanisms of MF in different stages of liver disease.

Liver Disease	Experimental Model	Molding Method	Dose	Core Mechanisms of Action and Targets	References
Acute liver injury	mice model	Combined treatment with lipopolysaccharide and D-galactosamine	20–100 mg/kg	↓TLR4, ↓NF-κB, ↓NLRP3, ↓TNF-*α*, ↓IL-1*β*, ↓caspase-1, ↑Nrf2, ↑HO-1, ↓ROS, ↓MDA	[74,76]
	mice model	Acetaminophen	no standards	↓APAP-Cys, ↓p-JNK, ↓ROS, ↑AMPK, ↑Nrf2, ↑HO-1	[83]
	mice model	CCl_4_	no standards	↑Nrf2, ↑NQO1, ↑HO-1, ↓p-p65	[80]
Fatty liver	mice model	High-fat diet feeding	10–50 mg/kg	↑AMPK*α*, ↓mTOR, ↓p-p70S6K, ↓LC3-II, ↓p62, ↓NF-κB, ↓TNF-*α*, ↓p-JNK, ↑PI3K, ↑p-AKT, ↓TG, ↓TC, ↓SREBP-1	[82]
Steatohepatitis	rat model	Alcohol gavage combined with a high-fat diet	20–80 mg/kg	↓NF-κB, ↓p65, ↓IL-6, ↓TNF-*α*, ↓SREBP-1c	[70,77]
Liver fibrosis	rat model	CCl_4_ subcutaneous injection	10–50 mg/kg	↓*α*-SMA, ↓COL1, ↓p-p65, ↓p-IkB *α*	[71,72,78]
Liver cirrhosis	mice model	CCl_4_ subcutaneous (2 mL/kg,10% olive oil solution) twice weekly for 12 weeks	50–100 mg/kg	↓EMT, ↓*α*-SMA, ↑E-cadherin, ↓p-JAK2, ↓p-STAT3, ↓TGF-*β*1	[71,73]
	mice model (BDL)	Bile duct ligation surgery with mancozeb intervention started 7 days postoperatively	100 mg/kg	↑DNase 2, ↓mtDNA, ↓TLR9, ↓MyD88, ↓NF-κB, ↑Nrf2, ↓ROS	[72]
	Rat model (alcohol)	Alcohol gavage (50% ethanol, 5 g/kg/d) in combination with a high-fat diet for 24 weeks	80–100 mg/kg	↑PPAR*α*, ↑PGC-1*α*, ↓HMGB1, ↓HSP90, ↓NLRP3	[70,78]
Liver cancer	rat model	Diethylnitrosamine induced	30–60 mg/kg	↑Bax, ↓Bcl-2, ↑Caspase-3, ↓NF-κB	[87]

↑—Upregulation, ↓—Downregulation.

## Data Availability

No new data were created or analyzed in this study. Data sharing is not applicable to this article.

## Abbreviations

The following abbreviations are used in this manuscript:

MF	Mangiferin
ALD	Alcoholic liver disease
MAFLD	Metabolism-associated fatty liver disease
Nrf2	nuclear factor E2-related factor 2
CCl_4_	carbon tetrachloride
HO-1	heme oxygenase-1
PPI	Protein–protein interaction
API	active pharmaceutical ingredients
NDV	Newcastle disease virus
